# Dysphagia in Huntington's Disease: An Evaluation of Swallowing Disturbance across the HD‐ISS Staging Spectrum

**DOI:** 10.1002/mdc3.70271

**Published:** 2025-08-07

**Authors:** Japleen Kaur, Krisha Bagga, Paul E. Gilbert, Jody Corey‐Bloom

**Affiliations:** ^1^ Neurosciences University of California San Diego San Diego California USA; ^2^ Department of Psychology San Diego State University San Diego California USA

**Keywords:** dysphagia, HD‐ISS staging system, Huntington's disease, swallowing impairment

## Abstract

**Background:**

The onset and pattern of progression of swallowing impairment in HD remain poorly understood. The Swallowing Disturbance Questionnaire (SDQ) has proven useful in assessing dysphagia across various neurological conditions, but its use in HD remains largely unexplored.

**Objectives:**

To evaluate swallowing difficulties in HD using the SDQ and Huntington's Disease Integrated Staging System.

**Methods:**

The SDQ was administered to 87 gene positive subjects stratified into HD‐ISS Stages 0/1 (*n* = 27), 2 (*n* = 21), and 3 (*n* = 39). Kruskal‐Wallis was used to compare performance among cohorts and partial eta‐squared (*η* ^2^) was used to calculate effect sizes. Spearman's rho (*r*) was used to calculate correlations.

**Results:**

Total swallowing disturbance score significantly differed across HD‐ISS stages (*ηp* ^2^ = 0.20, *p* < 0.001), with similar trends and strong effect sizes for both oral (*ηp* ^2^ = 0.15, *p* < 0.001) and pharyngeal (*ηp* ^2^ = 0.12, *p* < 0.001) phases. Within cohorts, total swallowing disturbance score was significantly different between Stages 0/1 and 2 (*p* = 0.007), Stage 2 and 3 (*p* = 0.02) and between Stages 0/1 and 3 (*p* < 0.001). Using quartile‐based severity cut‐points (Mild: 0–6, Moderate: 7–14, Severe: >14), 92.6% Stage 0/1 related mild swallowing problems, 7.4% of Stage 0/1 reporting moderate difficulties. In Stage 2, 28.5% described moderate and over 25% reported severe issues. Among Stage 3 subjects, one‐third reported moderate and almost half experienced severe swallowing difficulties.

**Conclusions:**

Swallowing disturbances emerge early and progressively worsen across the HD‐ISS staging spectrum, with both oral and pharyngeal phases affected. These findings suggest that early assessment of swallowing function is warranted in HD clinical care.

Huntington's disease (HD) is a devastating progressive neurodegenerative disorder inherited in an autosomal dominant pattern. The disease impacts four primary domains —behavioral, cognitive, motor, and functional abilities—significantly reducing quality of life for those with the mutation.^1^ Although early HD manifestations are predominantly behavioral, disease progression leads to severe cognitive and functional deterioration. Clinical diagnosis requires motor signs and symptoms consistent with HD, confirmed by a diagnostic confidence level of 4 (100% certainty) on the Unified Huntington's Disease Rating Scale (UHDRS) Total Motor Score (TMS).^2^

Dysphagia is defined as any abnormality in the transfer of a bolus from the mouth to the stomach.^3^ Swallowing is categorized into phases by the anatomical region in which it occurs: oral preparation phase, oral phase, pharyngeal and esophageal phase.^4^, ^5^ Swallowing impairment has been documented in HD patients across manifest disease stages, with one study reporting prevalence rates ranging from 35% in early stages to 100% in advanced stages using Dysphagia Outcome Severity Scale (DOSS).^5^ However, comprehensive characterization of dysphagia across the full HD disease spectrum remains limited. This complication can be life‐threatening, potentially causing aspiration pneumonia and malnutrition, and substantially diminishing quality of life as disease burden increases.^6^, ^7^

Currently, in clinical practice, swallowing evaluations are conducted using both instrumental assessment (including fiber‐optic endoscopic evaluation of swallowing and video fluoroscopic swallowing studies) and non‐instrumental clinical assessments, typically initiated once patients report symptoms that interfere with quality of life.^5^ However, there is considerable variability in clinical practice, with some institutions and speech‐language pathologists preferring modified barium swallow studies or clinical swallow evaluations as the initial assessment approach, highlighting the lack of standardized timing and methodology for early swallowing evaluation in HD. While it is known that swallowing disturbance worsens with disease progression in HD,^5^, ^8^, ^9^ critical knowledge gaps remain regarding precisely when these disturbances begin, and how they evolve across the various stages of HD. Furthermore, specific details concerning the nature of these swallowing disturbances, and their correlation with other manifestations in HD, remain limited.

A primary challenge lies in the inadequacy of traditional staging methods to characterize disease progression in HD. The UHDRS Total Motor Score (TMS), which assesses motor function, and Total Functional Capacity (TFC), which measures functional independence, have significant reliability issues.^10^ It has been suggested that subtle motor abnormalities are poor markers of HD phenoconversion, with low interrater reliability, leading to symptoms that are missed or misinterpreted.^11^ Notably, there is a conspicuous absence of swallowing assessment in both the UHDRS TMS and TFC. This omission makes it difficult to accurately evaluate swallowing impairment across different phases of HD using traditional staging methods in a cross‐sectional design.

To address these crucial gaps, our study utilizes Huntington's Disease Integrated Staging System (HD‐ISS) developed by Tabrizi et al^12^ to examine swallowing impairment across the various HD‐ISS stages. Unlike traditional staging (Shoulson‐Fahn staging system)^13^, which relies primarily on clinical observations, HD‐ISS incorporates biomarkers and imaging data to provide a more comprehensive and biologically driven assessment of disease progression.^14^ Stage 1 of the HD‐ISS captures the early neurodegeneration characterized by caudate and/or putamen atrophy, occurring before clear motor symptoms (Stage 2) and functional decline (Stage 3).^14^ Currently, we cannot pinpoint precisely when swallowing disturbance begins in HD and how it progresses in gene carriers stratified by HD‐ISS stage.

The SDQ has been previously validated against endoscopic evaluation for its use in HD.^15^ However, an established HD‐specific cut‐point for identifying dysphagia with the SDQ is lacking. Consequently, we currently rely on cut‐points adapted from Parkinson's disease (PD) and Multiple Sclerosis (MS) literature, which lack HD‐specific validation.^16^ Thus, existing reports exploring swallowing disturbance in HD with the SDQ have often employed a cut‐point of 11 derived from the PD and MS literature to classify an HD subject as having dysphagia. However, this approach carries the risk of overlooking subtle swallowing issues that fall below this threshold and may be clinically relevant. Therefore, a more inclusive approach is needed to capture early swallowing difficulties in HD.

To understand when swallowing disturbance begins and how it evolves in HD, we conducted the first cross‐sectional study using the SDQ across the HD‐ISS spectrum in a well‐characterized cohort of gene‐positive individuals. Our investigation specifically aimed to: (a) delineate the evolution of SDQ‐reported swallowing difficulties across the HD‐ISS stages; (b) determine how well these swallowing scores correlate with motor, cognitive, and functional measures on the UHDRS; and (c) develop an HD‐specific severity rating for the SDQ based on our gene‐positive cohort.

## Methods

### Ethics

This cross‐sectional study was conducted at the University of California, San Diego (UCSD) Huntington's Disease Society of America Centre of Excellence (HDSA CoE), following study approval by the UCSD Institutional Review Board (IRB) Committee (IRB Protocol #170038) and in accordance with the requirements of the Code of Federal Regulations on the Protection of Human Subjects.

### Participants

This cross‐sectional study was conducted over a period of 1 year. Eighty‐seven gene positive and fourteen normal control (NC) participants followed at the UCSD Huntington's Disease Society of America (HDSA) Center of Excellence (CoE) were recruited for this study. Inclusion criteria included age >18, CAG repeats >39, and ability to understand the English language well enough to respond to the SDQ questions. NC participants were recruited for visual comparison purposes and were not included in the statistical analyses. Informed consent was obtained from each participant prior to data collection.

### Assessments

#### Swallowing Disturbance Questionnaire (SDQ)

**Figure 1:**
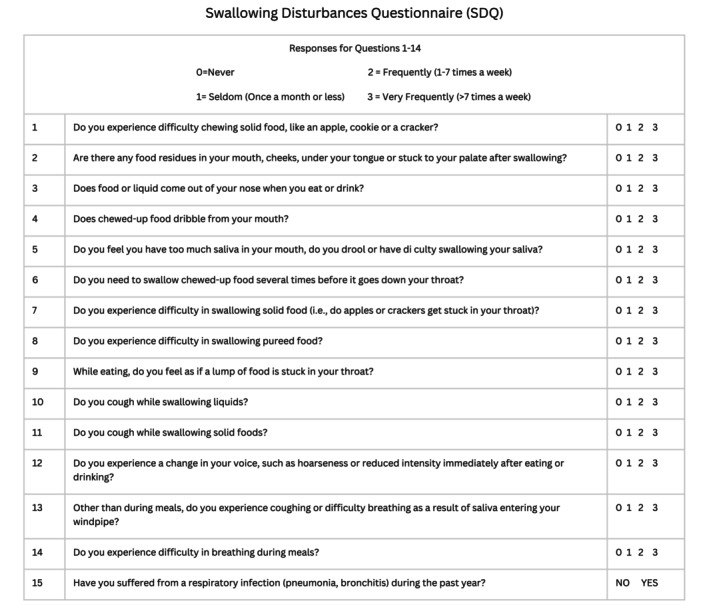
Swallowing disturbances questionnaire (SDQ).

Study participants, along with assistance from their caregivers, completed the SDQ, a 15‐item self‐report questionnaire focused on various aspects of swallowing disturbance (Fig. 1). The questions encompass common disturbances that may occur during the oral (Questions 1–5) and pharyngeal (Questions 6–14) phases of swallowing. These 14 questions are scored on a four‐point scale (0–3), in which 0 signifies no disturbance and 3 implies the frequent occurrence of symptoms throughout the day, indicative of severe disturbance. Question 15 is a “yes/no” question (yes scored as 2.5, no as 0). Total maximal score on the SDQ is 44.5.

#### Additional Clinical Assessments

Three additional clinical assessments from the UHDRS were administered to study participants: TFC, TMS, and the brief cognitive battery comprising the Symbol Digit Modality Test (SDMT), Stroop, and Verbal Fluency. In addition, participants were administered the Mini‐Mental State Examination (MMSE). The composite UHDRS (cUHDRS) score was also calculated for each participant.

### HD‐ISS Staging System

The HD‐ISS staging system is a staging system for HD research suggested by Tabrizi et al.^12^ Since volumetric MRI data was not available for the participants, we followed the recommended Prognostic Index Score (PIN) classification for HD‐ISS staging developed by Long and colleagues.^17^ Briefly, HD‐ISS stage 0 are participants who have the gene mutation only; Stage 1 are HD gene carriers with putamen and caudate atrophy; Stage 2 includes HD gene carriers with motor and/or cognitive, but no functional, difficulties; and Stage 3 are HD gene carriers who have functional decline as a result of their disease progression. In the absence of any clinical symptoms in Stages 0 and 1, for the purposes of this analysis, we elected to combine the two stages, as suggested by Long et al.^17^

### Statistical Analysis

Data were analyzed using GraphPad Prism 9.5.1 and SPSS Statistics for Macintosh. A Shapiro–Wilk test revealed that the data were not normally distributed (*p* < 0.01), so non‐parametric analyses were used. Initial group comparisons were conducted using Kruskal‐Wallis tests with Bonferroni correction. However, since age differed significantly across cohorts, further analyses were performed using Quade's non‐parametric analysis of covariance to control for age as a covariate. Partial eta‐squared (*η* ^2^) was used to calculate effect sizes. Spearman's rho (*r*) was used to calculate correlations between UHDRS measures and swallowing scores. Quartile analysis to categorize severity of the swallowing scores was conducted on Microsoft Excel.

## Results

Eighty‐Seven gene positive participants were classified into HD‐ISS stages: Stage 0/1 *N* = 27, Stage 2 *N* = 21 and Stage 3 *N* = 39. Consistent with HD progression, mean age showed a significant increase across the HD‐ISS stages; hence, was used as a co‐variate in the analyses. As expected, there was statistically significant cognitive decline with stage progression on the MMSE, SDMT, Categorical fluency, and Stroop Word (all *p* < 0.001). There was also an increase in the UHDRS TMS and Total Maximal Chorea score (TMC) with increase in disease stage (both *p* < 0.001). The buccal‐oral‐lingual chorea score (BOL chorea) showed significant differences across the staging spectrum (*p* = 0.001) as did the dysarthria and tongue protrusion (TP) component of the UHDRS motor examination (both *p* < 0.001) (Table 1).

**TABLE 1:** Mean (± standard deviation) demographics, for gene positive participants stratified by HD‐ISS staging

	Normal controls	Stage 0/1	Stage 2	Stage 3	*p*‐value*	Effect size (*η* ^2^)
*n*	14	27	21	39	‐	
Age(years)	60.77 (17.14)	39.18 (12.23)	52.85 (9.63)	55.23 (13.14)	<0.001	‐
Gender (M/F)	6/8	17/10	9/12	16/23	0.121	ns
MMSE	28.58 (2.82)	28.55 (1.31)	27.0 (2.34)	21.97 (7.63)	<0.001	0.19
SDMT	49.85 (11.58)	55.18 (9.71)	36.19 (10.90)	16.35 (10.31)	<0.001	0.48
Categorical fluency	20.54 (5.10)	24.61 (9.46)	16.47 (4.93)	9.16 (4.44)	<0.001	0.37
Stroop word	89.25 (17.37)	96.88 (22.34)	70.38 (17.70)	46.47 (18.80)	<0.001	0.35
TMS	0 (0)	1.66 (2.85)	12.38 (7.72)	45.15 (19.61)	<0.001	0.62
TMC	0 (0)	0 (0)	4.42 (3.26)	8.71 (4.91)	<0.001	0.36
BOL Chorea	0 (0)	0 (0)	0.38 (0.49)	1.10 (1.04)	0.0014	0.10
Dysarthria	0 (0)	0 (0)	0.23 (0.43)	1.28 (0.99)	0.000126	0.33
Tongue protrusion	0 (0)	0 (0)	0.23 (0.62)	1.56 (1.04)	<0.001	0.30
TFC	13 (0)	12.81 (0.55)	10.90 (2.04)	7.76 (3.41)	<0.001	0.23
Total swallowing disturbance score	0.57 (0.75)	1.37 (2.67)^a,c^	8.28 (7.34)^a,b^	13.12 (8.83)^b,c^	<0.001	0.20
Oral phase of swallowing	0.35 (0.49)	0.59 (1.44)^d,f^	2.34 (2.83)^d,e^	4.43 (3.29)^d,e,f^	<0.001	0.15
Pharyngeal phase of swallowing	0.21 (0.42)	0.81 (1.59)^g,h^	5.76 (5.32)^g^	8.75 (6.47)^h^	0.009	0.12

*Note*: Cohort performance compared using Quade's non‐parametric analysis of covariance with age as a covariate; Normal controls (*n* = 14) are shown for visual comparison but were not included in statistical analyses. Significant differences in the total swallowing disturbance scores between ^a^Stage 0/1 and Stage 2 (*p*‐value = 0.007); ^b^Stage 2 and Stage 3 (*p*‐value = 0.02); ^c^Stage 0/1 and Stage 3 (*p*‐value < 0.001). Significant differences in the Oral Phase of Swallowing between ^d^Stage 0/1 and Stage 2 (*p*‐value = 0.04); ^e^Stage 2 and Stage 3 (*p*‐value = 0.007); ^f^Stage 0/1 and Stage 3 (*p*‐value <0.001). Significant differences in the Pharyngeal Phase of Swallowing between ^g^Stage 0/1 and Stage 2 (*p*‐value = 0.01); ^h^Stage 0/1 and Stage 3 (*p*‐value <0.001).

Abbreviations: BOL chorea, Buccal‐oral‐lingual chorea; MMSE, Mini Mental State Examination; SDMT, Symbol Digit Modality Test; TFC, Total Functional Capacity; TMC, Total Maximal Chorea; TMS, Total Motor Score.

### Swallowing Evaluation

The total swallowing disturbance score was significantly different across the HD‐ISS staging spectrum with a strong effect size (*ηp* ^2^ = 0.20, *p* < 0.001). The oral and pharyngeal phases of swallowing also followed a similar trend with strong effect sizes of 0.15 and 0.12, respectively. Within cohorts, we saw significant differences between Stages 0/1 and 2 (*p* = 0.007), Stage 2 and 3 (*p* = 0.02) and between Stages 0/1 and 3 (*p* < 0.001) in the Total Swallowing Disturbance Score. For the oral phase of swallowing, there were significant differences between Stage 0/1 and Stage 2 (*p* = 0.04), Stage 2 and Stage 3 (*p* < 0.007) and between Stage 0/1 and Stage 3 (*p* < 0.001). In the pharyngeal phase of swallowing, significant differences were found between Stage 0/1 and Stage 2 (*p* = 0.01) and between Stage 0/1 and Stage 3 (*p* < 0.001) (Table 1).

Correlation analysis revealed significant associations between motor function and swallowing disturbances (Table 2). TMS demonstrated moderate to strong correlations with total swallowing disturbance score (*r* = 0.58, *p* < 0.01), oral phase score (*r* = 0.60, *p* < 0.01), and pharyngeal phase score (*r* = 0.60, *p* < 0.01). Similarly, TMC showed moderate correlations with total swallowing disturbance score (*r* = 0.50, *p* < 0.01) and pharyngeal phase score (*r* = 0.52, *p* < 0.01). Other motor parameters, including BOL chorea (*r* = 0.42, *p* < 0.01), TP (*r* = 0.53, *p* < 0.01), and dysarthria (*r* = 0.50, *p* < 0.01), also displayed significant correlations with total swallowing disturbance score.

**TABLE 2:** Correlations between motor, cognitive, and functional parameters and swallowing scores

Variables	Spearman's rho (*r*)	*p*‐value
TMS v Total Swallowing Disturbance Score	0.58	<0.01
TMS v Oral Phase of Swallowing	0.60	<0.01
TMS v Pharyngeal Phase of Swallowing	0.60	<0.01
TMC v Total Swallowing Disturbance Score	0.50	<0.01
TMC v Oral Phase of Swallowing	0.42	<0.01
TMC v Pharyngeal Phase of Swallowing	0.52	<0.01
BOL Chorea v Total Swallowing Disturbance Score	0.42	<0.01
BOL Chorea v Oral Phase of Swallowing	0.33	<0.01
BOL Chorea v Pharyngeal Phase of Swallowing	0.37	<0.01
Tongue Protrusion v Total Swallowing Disturbance Score	0.53	<0.01
Tongue Protrusion v Oral Phase of Swallowing	0.52	<0.01
Tongue Protrusion v Pharyngeal Phase of Swallowing	0.48	<0.01
Dysarthria v Total Swallowing Disturbance Score	0.50	<0.01
Dysarthria v Oral Phase	0.53	<0.01
Dysarthria v Pharyngeal Phase of Swallowing	0.53	<0.01
TFC v Total Swallowing Disturbance Score	−0.79	<0.01

*Note*: Spearman's rank correlation coefficients between clinical measures and swallowing scores.

Abbreviations: BOL, Buccal‐Oral‐Lingual chorea; TFC, Total Functional Capacity; TMC, Total Maximal Chorea; TMS, Total Motor Score.

Overall, out of the 87 gene‐positive subjects, nearly 40 percent (*n* = 34, 39.1%) reported clinically significant difficulty swallowing, as evidenced by a total SDQ score ≥11. This swallowing impairment was also reported by participants in the earlier disease stages: 4% of participants in Stage 0/1 and 47.6% in Stage 2 reported swallowing problems on the SDQ that resulted in scores of ≥11. This trend continued into Stage 3 where two‐thirds of participants described changes resulting in similarly elevated scores on the SDQ (Table 3).

**TABLE 3:** Total swallowing disturbance score stratified by PIN‐derived HD‐ISS stages *N* (%)

Stage	Total swallowing disturbance score ≤11	Total swallowing disturbance score ≥11
Stage 0/1 (*N* = 27)	26 (96.3)	1 (3.7)
Stage 2 (*N* = 21)	11 (52.4)	10 (47.6)
Stage 3 (*N* = 39)	16 (41.1)	23 (58.9)

In the absence of an established HD‐specific cut point for defining swallowing impairment on the SDQ, we sought to establish severity categories based on the quartile distribution of SDQ scores within our gene‐positive cohort. These quartile cut points were used to define Mild (scores encompassing the first two quartiles: 0–6); Moderate (scores within the third quartile: 7–14); and Severe (scores above the third quartile: >14) swallowing difficulties:

- SDQ scores 0–6: Mild swallowing difficulties
- SDQ scores 7–14: Moderate swallowing difficulties
- SDQ scores >14: Severe swallowing difficulties

Using these cut points, we found that 92.6% of Stage 0/1 participants reported mild swallowing difficulties and 7.4% of Stage 0/1 participants described moderate difficulties. More than a quarter of Stage 2 participants reported severe swallowing difficulties, which increased to nearly 50% in Stage 3 subjects (Table 4).

**TABLE 4:** Distribution of swallowing severity *N* (%) by stages

Stage	Mild swallowing difficulty (SDQ 0–6)	Moderate swallowing difficulty (SDQ 7–14)	Severe swallowing difficulty (SDQ > 14)
Stage 0/1 (*N* = 27)	25 (92.6)	7.4 (2)	0 (0)
Stage 2 (*N* = 21)	10 (47.6)	6 (28.5)	5 (23.9)
Stage 3 (*N* = 39)	8 (20.5)	13 (33.4)	18 (46.1)

## Discussion

To our knowledge, this is the first cross‐sectional study to specifically evaluate the SDQ across the spectrum of HD‐ISS stages in a well‐characterized cohort of HD gene‐positive individuals. We found that swallowing disturbance is an early feature of HD, often manifesting even prior to a formal motor diagnosis, and that swallowing difficulties only increase with each subsequent HD stage. This finding of early swallowing disruption builds upon previous results, such as the high prevalence of choking and swallowing difficulties reported by Aziz et al^18^ in the pre‐manifest stage of HD. Taken together, these results underscore the importance of evaluating and regular monitoring of early‐stage patients for subtle swallowing issues that may become more prominent as the disease progresses. In our study, the SDQ total swallowing disturbance score was found to correlate strongly with motor measures such as the TMS, and its components including TP, BOL chorea, and dysarthria. This is consistent with previous literature in which strong correlations were found between swallowing scores on the Dysphagia Outcome and Severity Scale^5^ and the UHDRS TMS, suggesting an association between worsening swallowing function and greater overall motor impairment.^19^ Finally, our data is supported by Kalkers et al,^20^ who found that swallowing dysfunction correlated strongly with the TFC, suggesting that worsening swallowing function was associated with declining independence.

Our analysis of swallowing ability across HD stages revealed a complex pattern of oral and pharyngeal involvement. Previous research, including Raines et al,^7^, ^19^ Kagel and Leopold,^21^ Pizzorni et al,^22^ and Schumann‐Werner et al,^23^ placed greater emphasis on oral, relative to pharyngeal, phase involvement in HD, while our findings suggest an evolution of impairment across the disease course, and a distinct shift in the relative severity of oral and pharyngeal dysfunction as HD progresses. In the earliest HD‐ISS stages (0/1), mean scores for the oral and pharyngeal phases were similar, indicating that both aspects of swallowing are likely affected to a comparable degree. This early, concurrent involvement of both oral and pharyngeal phases might reflect the initial impact of neurodegeneration on the motor systems that coordinate swallowing. As Raines et al^19^ highlighted, the oromotor system is vulnerable to the effects of HD‐related involuntary muscle contractions, and our data suggests that these early oromotor changes may disrupt both the oral propulsive functions, as well as the pharyngeal coordination necessary for swallowing. This is supported by the moderate‐strong correlations between oromotor components of the UHDRS—TP (*r* = 0.52); BOL chorea (*r* = 0.33); Dysarthria (*r* = 0.53); and the Total Swallowing Disturbance Score. It has been hypothesized that impaired initiation of the pharyngeal phase of swallowing, a critical component of swallowing, may be a consequence of atrophy of the caudate and putamen, a hallmark feature of Stage 1 in the HD‐ISS.^7^, ^8^, ^24^

In stages 2 and 3, on the other hand, we uncovered a shift in the pattern of severity impairment. Oral phase scores remained relatively stable (2.34 and 4.43, respectively), while pharyngeal phase scores showed a marked increase (5.76 and 8.75, respectively), suggesting a worsening of pharyngeal relative to oral dysfunction. The increased severity of pharyngeal impairment in later stages may have significant clinical implications, potentially contributing to an elevated risk of aspiration and other respiratory complications. Pizzorni et al also found a correlation between disease stage and delayed pharyngeal phase.^22^ Considering the established link between silent aspiration (caused by diminished sensorimotor response to food/fluids passing below the level of the vocal folds and into the airway)^21^, ^23^, ^24^, ^25^ and the development of aspiration pneumonia,^25^ the pharyngeal dysfunction we have identified early in the course of the disease underscores the need for further research into long‐term swallowing safety across all stages of HD.

The mean SDQ score of our Stage 3 cohort (13.12) was remarkably similar to the mean SDQ score^15^, ^26^ reported by Manor et al in their study of 14 HD participants. However, their definition of dysphagia relied on the SDQ cut point of 11 derived from PD,^15^ a condition with distinct underlying pathophysiology affecting swallowing. While both diseases involve neurodegeneration, the specific patterns of motor and cognitive involvement differ significantly. For instance, the prominent rigidity and bradykinesia in PD, often leading to hypokinetic dysarthria and slowed swallowing initiation,^26^, ^27^ contrasts significantly with the complex movement profile in HD, involving both hyperkinetic movements (early‐moderate stages) and hypokinetic features (later stages), which have been associated with the emergence of dysphagia in HD.^22^, ^28^ This fundamental difference in primary motor impairment contributing to swallowing difficulties in the two conditions further underscores the potential inadequacy of a universal cut point for swallowing difficulty, and the need for an HD‐specific value.

Our proposed severity rating system, classifying participants into mild, moderate, and severe swallowing difficulties, addresses the absence of a validated HD‐specific cut point for the SDQ. The notable proportion of individuals in early HD stages exhibiting mild to moderate swallowing issues underscores the potential for standard clinical examination to miss these subtle impairments. This system therefore carries significant clinical implications, potentially enabling the identification of early swallowing problems in individuals with the HD gene even before overt dysphagia manifests. Existing evidence suggests that early detection, and intervention, can lead to improved long‐term swallowing outcomes in this population.^22^ Furthermore, HD‐specific thresholds may offer a more accurate measure of disease related swallowing impairment and facilitate enhanced monitoring of disease progression and response to potential therapies. A self‐reported tool like the SDQ combined with objective functional assessments such as the Timed Water Swallow Test (TWST) and/or Test of Mastication and Swallowing Solids (TOMASS) may represent best practice for clinical monitoring of dysphagia progression in HD.

While our study is among the largest to date to examine self‐reported swallowing disturbances across the various stages of HD in a gene‐positive cohort, it is not without limitations. Primarily, the cross‐sectional design of our study precludes the examination of longitudinal changes in swallowing presentation across HD stages. Future research should prioritize longitudinal studies to confirm and expand upon these findings. These investigations should integrate Fiberoptic Endoscopic Evaluation of Swallowing (FEES) and Videofluoroscopic Swallowing Study (VFSS) with the SDQ, especially in the early stages of HD, to provide objective validation of pharyngeal and oral findings that may be subtle and potentially overlooked during clinical examination alone. Additionally, the HD‐specific thresholds proposed in this study warrant validation through objective instrumental assessment of swallowing mechanics. In summary, our study significantly expands the current understanding of dysphagia in HD by systematically characterizing its evolution across the HD‐ISS staging spectrum. Our findings demonstrate that swallowing impairment is evident even in the early stages of HD and progressively worsens with increasing disease stage. Notably, both oral and pharyngeal phases exhibit early signs of dysfunction, however, the pharyngeal phase demonstrates a more pronounced acceleration of decline in later stages. Furthermore, we addressed a key limitation in the literature—the absence of an HD‐specific cut point—by developing severity thresholds for the SDQ to facilitate the identification of early swallowing difficulties that may be missed by standard clinical interview.

## Author Roles

(1) Research project: A. Conception, B. Organization, C. Execution. (2) Statistical Analysis: A. Design, B. Execution, C. Review and Critique. (3) Manuscript Preparation: A. Writing of the first draft, B. Review and critique.

J.K.: 1A, 1B, 1C, 2A, 2B, 2C, 3A.

K.B.: 1B, 1B, 2C, 3B.

P.G.: 1A, 2C, 3B.

J.C.B.: 1A, 2A, 2B, 2C, 3B.

## Disclosures

**Ethical Compliance Statement:** Institutional review board approval was obtained from the Institutional review board of the University of California, San Diego (Protocol no. #170038). Written informed consent was obtained from all participants before participation. We confirm that we have read the Journal's position on issues involved in ethical publication and affirm that this work is consistent with those guidelines.

**Funding Sources and Conflicts of Interest:** No specific funding was received for this work. The authors declare that there are no conflicts of interest relevant to this work.

**Financial Disclosures for the Previous 12 Months**: The authors declare that there are no financial disclosures for the previous 12 months.

## Data Availability

The data that support the findings of this study are available from the corresponding author upon reasonable request.
